# Dietary Magnesium Intake Is Associated With Self‐Reported Short Sleep Duration but Not Self‐Reported Sleep Disorder

**DOI:** 10.1002/brb3.70251

**Published:** 2025-02-05

**Authors:** Shuhua Zhao, Jingping Hu, Chuannan Yue, Jingling Tian, Shaoli Zhou, Qianqian Zhu

**Affiliations:** ^1^ Department of Anesthesiology The Seventh Affiliated Hospital of Sun Yat‐Sen University Shenzhen City People's Republic of China; ^2^ Department of Anesthesiology The Third Affiliated Hospital of Sun Yat‐Sen University Guangzhou City People's Republic of China

**Keywords:** self‐reported sleep duration, self‐reported sleep disorders, magnesium

## Abstract

**Background:**

Sleep disturbances have become increasingly prevalent in modern society. Research suggests that a deficiency in magnesium (Mg) may contribute to sleep disturbances. This study aims to investigate the association between daily Mg intake and self‐reported sleep duration and sleep disorders using data from the National Health and Nutrition Examination Survey (NHANES).

**Methods:**

The study dataset includes participants from five cycles (2009–2018) of NHANES. The associations between Mg intake and sleep duration are analyzed using weighted logistic regression.

**Results:**

Baseline characteristics of 21,840 participants were analyzed. Mg intake was independently associated with sleep duration (OR = 1.07, 95% CI (1.01–1.14), *p* = 0.024). Higher quartiles of Mg intake from food were associated with normal sleep duration. However, Mg intake from food in participants with self‐reported sleep disorders did not differ from those without sleep disorders (OR = 0.96, 95% CI (0.90–1.03), *p* = 0.238). Among 3923 participants with Mg supplementation data, no significant differences were found between the top and bottom 50% Mg supplementary groups regarding sleep duration or disorders.

**Conclusions:**

Dietary Mg intake is independently associated with self‐reported short sleep duration but not with self‐reported sleep disorders. Mg supplementation is not associated with either self‐reported sleep duration or sleep disorders.

## Graphical abstract

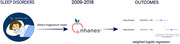

## 1 Introduction

Sleep is a vital physiological process that is crucial for overall health and well‐being. Adequate and high‐quality sleep plays a critical role in various aspects of human functioning, including cognitive performance, physical health, and emotional well‐being (Palagini et al. 2022). However, sleep disturbances, such as short sleep duration and poor sleep quality, have become increasingly common in modern society, posing significant challenges to public health (Baranwal, Yu, and Siegel 2023). Therefore, it is crucial to understand the factors that influence sleep and explore potential interventions to improve sleep quality.

Magnesium (Mg), an essential micronutrient, has been implicated in the regulation of sleep. It plays a vital role in several physiological processes, including neurotransmitter synthesis, muscle relaxation, and the regulation of the sleep–wake cycle (Chollet et al. 2001; Hornyak et al. 1998). Previous research has suggested that a deficiency in Mg may contribute to sleep disturbances, such as difficulty falling asleep and frequent awakenings during the night (Nielsen, Johnson, and Zeng 2010). Conversely, increasing Mg intake has been proposed as a potential strategy to enhance sleep quality and duration (Abbasi et al. 2012).

To investigate the potential association between daily Mg intake and self‐reported sleep duration and sleep disorders, this study aims to analyze data from the National Health and Nutrition Examination Survey (NHANES).

## 2 Methods

### 2.1 Database and Sample

The study dataset includes participants from five cycles (2009–2018) of the NHANES. Detailed information about the participants’ recruitment procedure and data collection can be found at https://www.cdc.gov/nchs/nhanes/index.htm. The NHANES study protocol was approved by the Research Ethics Review Board of the National Center for Health Statistics, and all participants provided written informed consent.

After excluding participants who were pregnant at screening and those with missing sleep variable and dietary Mg records, a total of 21,840 participants aged 19 years and older were included in the present study.

### 2.2 Variables and Definitions

The database included two questions about sleep: “Usual sleep time on weekdays or workdays” and “Ever told doctor had trouble sleeping?” The “Usual sleep time on weekdays or workdays” considered as sleep hours and categorized as short (< 7 h per night) or normal (7 h or more) based on previous studies and recommendations from the American Academy of Sleep Medicine and Sleep Research Society (Grandner et al. 2010; Watson et al. 2015). If participants answered “yes” to the question “Ever told doctor had trouble sleeping?,” it was defined as “self‐reported sleep disorder.”

The Mg intake data were obtained from the dietary intake data, which estimated the types and amounts of foods and beverages consumed (including all types of water). The first dietary recall interview was conducted in‐person at the Mobile Examination Center (MEC), and the second interview was conducted via telephone 3–10 days later. The supplement data were collected from the 24‐h dietary supplement use component, which gathered information on the types and amounts of supplements consumed during the 24‐h period prior to the interview (midnight to midnight).

A 2‐day dietary intake in NHANES was using the National Cancer Institute method to estimate the total Mg, calcium, vitamin D, and caffeine intakes from “food only” and from “supplement” (Ahluwalia et al. 2016).

Other variables included in the analysis were age, gender, body mass index (BMI), hypertension, diabetes, marital, race, education, ratio family income to poverty (INDFMPIR), cigarette smoking, alcohol consumption, general health condition, work activity, and recreational activities. Hypertension was defined as a response to the question “Have you ever been told by a doctor or other health professional that you have hypertension also called high blood pressure?” Diabetes was defined as a positive to the question “Have you ever been told by a doctor or health professional that you have diabetes or sugar diabetes?”

### 2.3 Statistical Analysis

Data were presented as proportions, means, and standard errors. The statistical analysis included student's *t*‐tests for continuous variables and chi‐square tests for categorical variables. Since the data were collected from five cycles, all samples were weighted accordingly during analysis.

The relationship between Mg intake and sleep patterns was explored using weighted logistic regression models. In these regression analyses, the dietary intake data were categorized into quartiles or divided into top fifty percent and low fifty percent groups. Additional regression analyses were conducted to adjust for covariates, including age, gender, BMI, hypertension, diabetes, marital status, race, education level, INDFMPIR, cigarette smoking, alcohol consumption, general health condition, work activity, and recreational activities. The dietary intake data were also included in these regression analyses. All statistical analyses were performed using R software, version 4.3.2 (R Foundation for Statistical Computing, Vienna, Austria; http://www.r‐project.org accessed on May 1, 2023), and a *p*‐value less than 0.05 was considered statistically significant.

## 3 Results

A total of 21,840 participants were included in the present analyses.

### 3.1 Self‐Reported Short Sleep

Out of the participants, 7324 (33.53%) had short sleep duration. Significant differences were observed between participants with short and normal sleep duration in terms of age, gender, BMI, hypertension, diabetes, marital status, race, education level, INDFMPIR, cigarette smoking, alcohol consumption, general health condition, work activity, and recreational activities (all *p* < 0.001, Table 1).

**TABLE 1:** Comparisons between self‐reported short and normal sleep duration.

Variables	< 7 h (7324) short	≥7 h (14516) normal	*p*
Age (years)	47.37 ± 15.62	48.92 ± 17.40	< 0.001
Female, *n* (%)	3559 (48.59%)	7782 (53.61%)	< 0.001
BMI (kg/m^2^)	29.94 ± 7.29	29.00 ± 6.76	< 0.001
Marital status			< 0.001
Married/cohabiting, *n* (%)	4509 (61.56%)	9418 (64.88%)	
Widowed/divorced/separated, *n* (%)	1495 (20.41%)	2537 (17.48%)	
Never married, *n* (%)	1320 (18.03%)	2561 (17.64%)	
Race			< 0.001
Mexican American, *n* (%)	593 (8.09%)	1150 (7.92%)	
Other Hispanic, *n* (%)	495 (6.76%)	762 (5.25%)	
Non‐Hispanic White, *n* (%)	4495 (61.38%)	10237 (70.52%)	
Non‐Hispanic Black, *n* (%)	1193 (16.29%)	1252 (8.63%)	
Other Race, *n* (%)	548 (7.48%)	1115 (7.68%)	
Education lever			< 0.001
Less than 9th grade, *n* (%)	333 (4.54%)	643 (4.43%)	
Ninth‐11th grade, *n* (%)	800 (10.92%)	1259 (8.67%)	
No less than high school graduate, *n* (%)	6191 (84.54%)	12614 (86.90%)	
Family income to poverty	2.85 ± 1.58	3.07 ± 1.59	< 0.001
Hypertension, *n* (%)	2552 (34.84%)	4671 (32.18%)	< 0.001
Diabetes, *n* (%)	813 (11.10%)	1450 (9.99%)	< 0.001
General health			< 0.001
Good/vary good/excellent, *n* (%)	5488 (74.93%)	11748 (80.93%)	
Fair, *n* (%)	1257 (17.16%)	1893 (13.04%)	
Poor or other, *n* (%)	579 (7.91%)	875 (6.03%)	
Vigorous/moderate work activity	1.20 ± 0.45	1.23 ± 0.45	< 0.001
Vigorous/moderate recreational activity	1.23 ± 0.48	1.25 ± 0.50	< 0.001
Smoke, *n* (%)	1444 (19.71%)	1781 (12.27%)	< 0.001
Drink, *n* (%)	1820 (24.85%)	4076 (28.08%)	< 0.001
Caffeine (mg)	177.70 ± 208.87	162.90 ± 169.51	< 0.001
Vitamin D (mcg)	4.61 ± 4.55	4.72 ± 4.53	< 0.001
Calcium (mg)	962.90 ± 524.60	969.92 ± 490.25	< 0.001
Potassium (mg)	2649.70 ± 1110.31	2692.96 ± 1083.48	< 0.001
Magnesium (mg)	297.39 ± 135.07	306.54 ± 131.59	< 0.001

Abbreviation: BMI: body mass index.

The dietary intake of potassium, and Mg from food only was significantly lower in participants with short sleep duration compared to those with normal sleep duration (all *p* < 0.001, Table 1).

**FIGURE 1:**
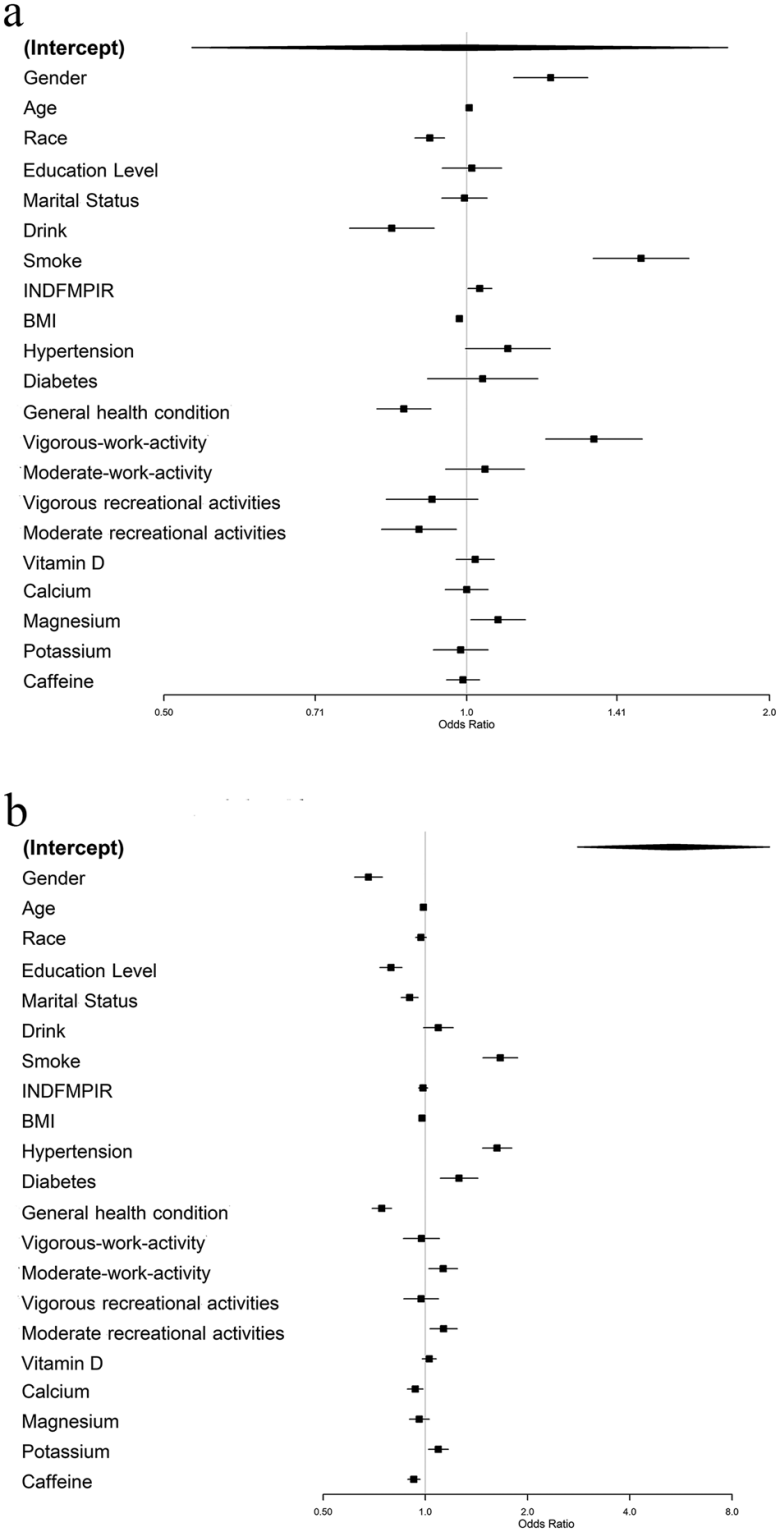
Regression results of the association between factors and self‐reported sleep duration (a) or sleep disorder (b).

Weighted logistic regression analysis revealed that Mg intake was independently associated with sleep duration (OR = 1.07, 95% CI (1.01–1.14), *p* = 0.024, Figure 1a). The dietary intake of caffeine was not independently associated with sleep duration after weighted logistic regression analysis (Figure 1a).

**FIGURE 2:**
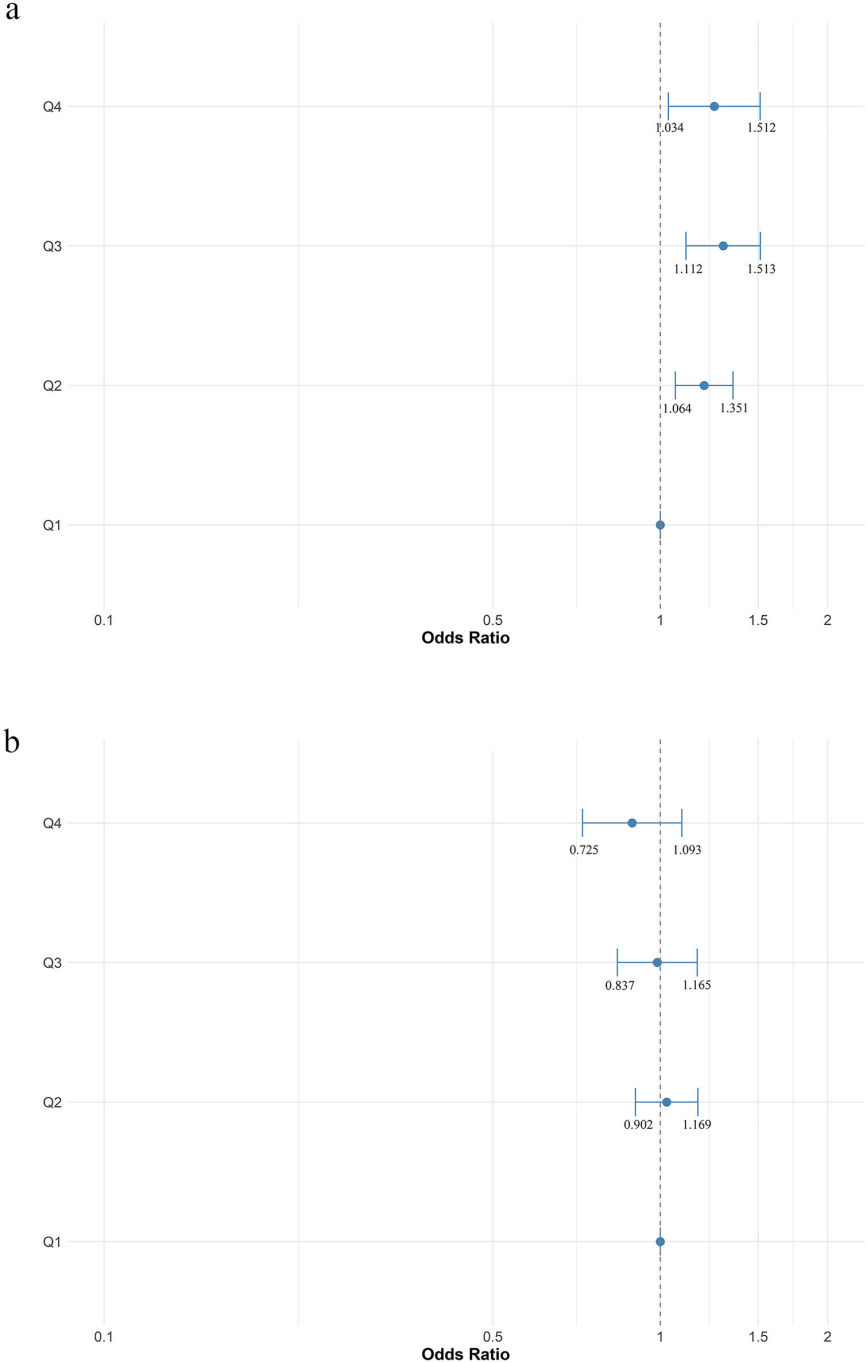
Multivariable‐adjusted ORs and 95% CIs of normal self‐reported sleep duration (a) or sleep disorder (b) by quartiles of magnesium intake levels.

After adjusting for age, gender, BMI, hypertension, diabetes, marital status, race, education level, INDFMPIR, cigarette smoking, alcohol consumption, general health condition, work activity, recreational activities, caffeine, vitamin D, calcium, and potassium, participants in the higher quartile of Mg intake from food were associated with normal sleep duration (*p* trend = 0.024, Figure 2a).

### 3.2 Self‐Reported Sleep Disorder

A total of 5965 (27.31%) participants reported sleep disorder. Significant differences were observed in the characteristics between participants with and without self‐reported sleep disorder (all *p* < 0.001, Table 2). However, there was no significant difference in Mg intake from food between participants with and without self‐reported sleep disorder (OR = 0.96, 95% CI (0.90–1.03), *p* = 0.238, Figure 1b). Weighted logistic regression analysis also showed no association between Mg intake from food and self‐reported sleep disorder (*p* trend = 0.238, Figure 2b).

**TABLE 2:** Comparisons between with and without self‐report sleep disorder.

Variables	Yes (5965)	No (15875)	*p*
Age (years)	51.96 ± 15.58	47.01 ± 17.20	< 0.001
Female, *n* (%)	3518 (58.97%)	7820 (49.26%)	< 0.001
BMI (kg/m^2^)	30.44 ± 7.67	28.81 ± 6.55	< 0.001
Married status			< 0.001
Married/cohabiting, *n* (%)	3597 (60.31%)	10374 (65.35%)	
Widowed/divorced/separated, *n* (%)	1477 (24.76%)	2499 (15.74%)	
Never married, *n* (%)	891 (14.93%)	3002 (18.91%)	
Race			< 0.001
Mexican American, *n* (%)	285 (4.78%)	1473 (9.28%)	
Other Hispanic, *n* (%)	247 (4.14%)	1008 (6.35%)	
Non‐Hispanic White, *n* (%)	4464 (74.83%)	10295 (64.85%)	
Non‐Hispanic Black, *n* (%)	604 (10.13%)	1791 (11.28%)	
Other Race, *n* (%)	365 (6.12%)	1308 (8.24%)	
Education lever			< 0.001
Less than 9th grade, *n* (%)	211 (3.54%)	770 (4.85%)	
Ninth‐11th grade, *n* (%)	552 (9.25%)	1491 (9.39%)	
No less than high school graduate, *n* (%)	5202 (87.21%)	13614 (85.76%)	
Family income to poverty	2.97 ± 1.62	3.01 ± 1.58	
Hypertension, *n* (%)	2709 (45.42%)	4424 (27.87%)	< 0.001
Diabetes, *n* (%)	896 (15.02%)	1332 (8.39%)	< 0.001
General health			< 0.001
Good/vary good/excellent, *n* (%)	4281 (71.77%)	13040 (82.14%)	
Fair, *n* (%)	1203 (20.17%)	1884 (11.87%)	
Poor or other, *n* (%)	481 (8.06%)	951 (5.99%)	
Vigorous/moderate work activity	1.24 ± 0.46	1.21 ± 0.44	< 0.001
Vigorous/Moderate recreational activity	1.27 ± 0.49	1.23 ± 0.50	
Smoke, *n* (%)	1107 (18.55%)	2042 (12.86%)	< 0.001
Drink, *n* (%)	1760 (29.51%)	4145 (26.11%)	< 0.001
Caffeine (mg)	182.96 ± 194.36	160.96 ± 176.89	< 0.001
Vitamin D (mcg)	4.58 ± 4.55	4.73 ± 4.53	< 0.001
Calcium (mg)	946.98 ± 489.17	976.36 ± 505.37	< 0.001
Potassium (mg)	2601.92 ± 1040.80	2711.94 ± 1110.55	< 0.001
Magnesium (mg)	296.06 ± 130.72	306.95 ± 133.40	< 0.001

Abbreviation: BMI: body mass index.

### 3.3 Mg Supplementary and Sleep

**FIGURE 3:**
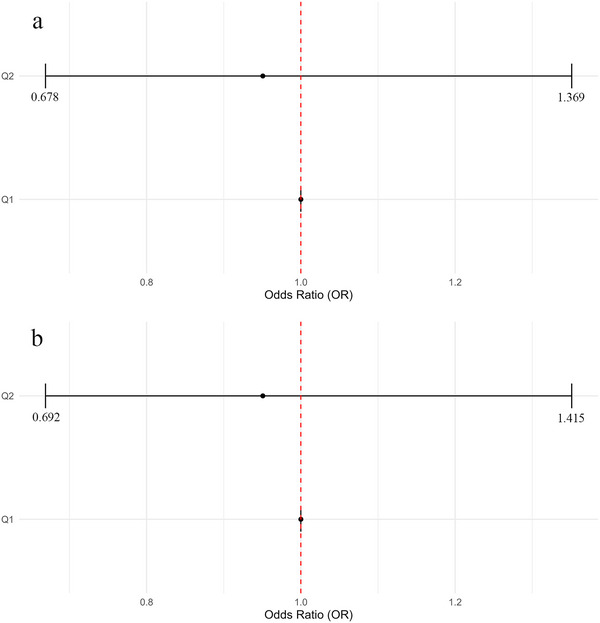
Multivariable‐adjusted ORs and 95% CIs of normal self‐reported sleep duration (a) or sleep disorder (b) by fifty‐fifty of magnesium supplementary levels.

Only 3923 participants had Mg supplementary data. The Mg supplementary data were divided into top fifty percent and low fifty percent groups since the first and second quartile cutoff values were the same. Weighted logistic regression analysis showed no significant difference in self‐reported sleep duration or sleep disorder between participants in the top fifty percent and low fifty percent Mg supplementary groups (Figure 3).

## 4 Discussion

The present study demonstrates that dietary intake of Mg from food is only associated with self‐reported sleep duration, not sleep disorders. Compared to those with lower Mg intake, individuals with higher Mg intake from food have a higher likelihood of having a normal sleep duration, even after adjusting for other variables. However, the study found no association between Mg supplementation and either self‐reported sleep duration or sleep disorders.

Insufficient sleep is a prevalent issue in developed countries, including the United States (Roenneberg 2013). It is estimated that 50–70 million Americans suffer from chronic sleep disorders (Institute of Medicine Committee on Sleep and Research 2006). The present study reveals that approximately one‐third of the US population reports short sleep duration, while a quarter of the population reports sleep disorders. Sleep disorders have been linked to various physical and psychological problems. Caffeine consumption is a common dietary factor believed to be associated with sleep (Gardiner et al. 2023). Interestingly, the present study suggested that dietary caffeine serves as a “protective factor” for self‐reported sleep disorders, although it was not linked to sleep duration. However, the specific form and timing of caffeine intake were not clearly documented in the NHANES database. Additionally, the relationship between caffeine and insomnia symptoms is influenced by sleep duration, with sleep disorders being inversely associated with sleep duration (Chaudhary et al. 2016). Calcium and vitamin D have been shown to be associated with sleep in prior research (Romano et al. 2020; Zhang et al. 2022). The present study found that only vitamin D was associated with self‐reported sleep disorders.

Research has shown that individuals who sleep less than 7 h per night are more prone to burnout compared to those who sleep more than 7 h (Saintila et al. 2023). Additionally, in patients without metabolic syndrome, short sleep duration has been associated with an increased risk of stroke (Wu et al. 2023). The USA National Sleep Foundation recommends that adults aim for 7–9 h of sleep per night (Hirshkowitz et al. 2015). Similarly, the US National Centers for Disease Control and Prevention considers a sleep duration of 7 h or more as healthy, based on the Behavioral Risk Factor Surveillance System (Liu et al. 2016). Achieving 7 h of sleep is one of the recommendations for maintaining good sleep hygiene (Baranwal, Yu, and Siegel 2023).

Mg is an essential mineral that acts as a cofactor in over 300 enzyme systems (Schwalfenberg and Genuis 2017). Previous studies have shown that Mg is involved in the regulation of cellular timekeeping, energy balance, and sleep electroencephalogram (EEG) patterns (Feeney et al. 2016; Held et al. 2002). Mg deficiency has also been explored in various clinical conditions, including sleep disorders (Arab et al. 2023; Han et al. 2024; Serefko, Szopa, and Poleszak 2016). For example, a study on healthy elderly individuals found that lower blood Mg levels were associated with shorter sleep duration and increased telomere attrition rate (Dhillon et al. 2023). However, another study on older outpatients found that serum Mg levels were only related to daytime sleepiness and not insomnia (Tunc et al. 2023). It is important to note that blood Mg levels may not accurately reflect total Mg levels, as they account for less than 1% of the total (Kielstein and David 2013; Schwalfenberg and Genuis 2017). Additionally, blood Mg levels are influenced by genetic factors, whereas brain Mg levels have been shown to be unrelated to genetics but correlated to sleep quality in animal models (Chollet et al. 2001, 2000).

In addition to blood Mg levels, previous studies have also explored the potential association between dietary Mg intake and sleep. A 5‐year‐up cohort study in China found that higher dietary Mg was associated with a lower likelihood of daytime sleepiness, but only in female adults (Cao et al. 2018). On the other hand, researchers from Japan and another study did not find an association between self‐reported dietary Mg intake and sleep duration or inadequate Mg intake and short sleep, respectively (Ikonte et al. 2019; Komada et al. 2017). A recent systematic review of 7582 subjects found that observational studies, but not randomized clinical trials, showed an association between Mg consumption (either through high dietary intake or supplementation) and sleep quality (Arab et al. 2023). The present study demonstrates that dietary Mg intake is only associated with self‐reported sleep duration but not sleep disorders. Further research is needed to better understand the role of Mg in sleep regulation and its potential implications for sleep.

Due to the potential role of Mg in sleep regulation, several studies have investigated the effects of Mg supplementation on sleep disorders. In patients after heart surgery, Mg supplementation was found to improve sleep quality as assessed by the Petersburg Sleep Quality Inventory (PSQI) (Saba et al. 2022). However, Mg supplementation did not have any effect on duration in elderly individuals (Abbasi et al. 2012). A meta‐analysis also failed to find evidence supporting the use of oral Mg supplementation for the treatment of insomnia in adults (Mah and Pitre 2021). In line with these findings, the present study demonstrates that participants who took oral Mg supplementation did not show any significant differences in self‐reported sleep duration or sleep disorders compared to those who did not take supplements. However, considering that Mg supplementation is relatively inexpensive and easily accessible, oral Mg supplements could be worth trying (Mah and Pitre 2021). Legumes, seeds, nuts, whole‐grain cereals, and leafy vegetables are excellent sources of Mg (https://fdc.nal.usda.gov/food‐search?component=1090). Consequently, incorporating these foods into the diet on a regular basis can help adults meet the recommended intake of Mg. Although the recommended dietary allowance for Mg is more than 300 mg for adults, about half of Americans consume less than the estimated average requirement (Costello et al. 2016; Schwalfenberg and Genuis 2017).

Several limitations of the present study should be acknowledged. First, dietary and sleep data from NHANES were obtained through recall interviews, which introduces the possibility of recall bias. Second, the database does not provide information on the sources of caffeine, calcium, vitamin D, and Mg intake, nor does it specify the timing of their consumption. It does not clarify whether these factors are related to sleep. Third, while the study explored several potential factors influencing sleep, including caffeine consumption, calcium intake, overall health status, vigorous physical activity, and recreational activities, other relevant factors—such as somatic diseases and individual lifestyle choices like night shifts—were not considered. As a result, the findings of this study warrant further validation through prospective research.

## 5 Conclusion

The present study suggests that dietary Mg intake is independently associated with self‐reported short sleep duration but not self‐reported sleep disorders. However, Mg supplementation may not have any significant association with either self‐reported sleep duration or sleep disorders.

## Author Contributions

**Shuhua Zhao**: software, data curation, formal analysis, writing–review and editing. **Jingping Hu**: software, data curation, formal analysis, writing–review and editing. **Chuannan Yue**: software, data curation, writing–review and editing, formal analysis. **Jingling Tian**: data curation, writing–review and editing. **Shaoli Zhou**: conceptualization, software, methodology, formal analysis, supervision, writing–review and editing. **Qianqian Zhu**: conceptualization, methodology, software, formal analysis, supervision, funding acquisition, writing–original draft, writing–review and editing.

## Ethics Statement

The NHANES study protocol was approved by the Research Ethics Review Board of the National Center for Health Statistics and all participants provided written informed consent.

## Conflicts of Interest

The authors declare no conflicts of interest.

### Peer Review

The peer review history for this article is available at https://publons.com/publon/10.1002/brb3.70251.

## Data Availability

The datasets supporting the conclusions of this article are available in the NHANES repository [unique persistent identifier and hyperlink to datasets in https://www.cdc.gov/nchs/nhanes/index.htm].
